# Relationship between mental health literacy and professional psychological help-seeking attitudes in China: a chain mediation model

**DOI:** 10.1186/s12888-023-05458-5

**Published:** 2023-12-21

**Authors:** Jingyuan Yang, Yunjia Li, Rui Gao, Hui Chen, Zhihui Yang

**Affiliations:** 1 Department of Psychology, School of Humanities and Social Sciences, Beijing Forestry University, 35 Tsinghua East Road, Haidian District, Beijing, 100083 P. R. China; 2 Center for Strategic Studies, Qiyuan Laboratory, Building 1st, Yard 58th, Yinhua Road, Zhongguancun Environmental Protection Science and Technology Park, Haidian District, Beijing, 100095 P. R. China

**Keywords:** Distress disclosure, Mental health literacy, Professional psychological help-seeking attitudes, Psychological help-seeking stigma

## Abstract

**Background:**

Mental health literacy is considered an important factor in promoting professional psychological help-seeking attitudes. However, it is important to clarify the emotional and behavioral factors that underlie this association. Drawing from the ABC model of attitudes and the Health Disclosure Decision-Making Model, this study aimed to examine the mediating role of psychological help-seeking stigma and distress disclosure in the relationship between mental health literacy and professional psychological help-seeking attitudes.

**Methods:**

We collected data from 827 participants in seven regions of China (597 female; *M*_age_ = 26.019, *SD* = 5.592 years old) using self-report measures, including the Mental Health Literacy Scale, Questionnaire of Stigma for Seeking Professional Psychological Help, Distress Disclosure Index, and Attitudes Toward Seeking Professional Psychological Help-Short Form. A chain mediation model was built to examine the relationships among mental health literacy, psychological help-seeking stigma, distress disclosure, and professional psychological help-seeking attitudes.

**Results:**

The results of the analyses showed a positive association between mental health literacy and professional psychological help-seeking attitudes, with psychological help-seeking stigma and distress disclosure playing mediating roles in this relationship. Furthermore, even after controlling for participants’ age, gender, and education, the chain mediation effect of psychological help-seeking stigma and distress disclosure on the relationship between mental health literacy and professional psychological help-seeking attitudes was observed among the Chinese population.

**Conclusions:**

These findings underscore the significance of mental health literacy in shaping professional psychological help-seeking attitudes, while emphasizing the need to account for psychological help-seeking stigma and distress disclosure when examining this association. Additionally, the model proposed in this study provides a valuable framework for promoting the utilization of professional mental health services.

## Introduction

In China, the lifetime prevalence rate of various mental illnesses among adults over 18 years old is 16.57% [1]. If left untreated, mental health disorders can worsen over time and have a significant impact on individuals’ quality of life [2–4], leading to adverse occupational outcomes such as unemployment, sick leave, and job loss [5], and increased risk of suicidal behavior [6–8]. These adverse outcomes associated with mental health disorders highlight the crucial role of seeking professional psychological help.

However, over 91% of individuals with mental disorders have not received adequate treatment [9]. Previous research found that professional psychological help-seeking attitudes were closely associated with mental health service utilization [10–13]. Professional psychological help-seeking attitudes refers to the cognitive, emotional, and behavioral tendencies toward professional psychological help-seeking behavior when individuals experience psychological problems or diseases [14]. In order to decrease the possibility resulting from untreated mental health disorders, it is therefore significant to clarify the factors and inter mechanisms associated with professional psychological help-seeking attitudes.

### Mental health literacy and professional psychological help-seeking attitudes

One factor which has been indicated to be associated strongly with professional psychological help-seeking attitudes is mental health literacy. Mental health literacy encompasses knowledge and beliefs that aid in recognizing, managing, and preventing mental disorders [15]. According to the ABC model of attitudes (also known as *The tripartite model of attitudes*; A refers to “Affective components”; B refers to “Behavioral components”; C refers to “Cognitive components”), cognitive factors such as mental health literacy play a crucial role in shaping attitudes [16, 17]. Numerous studies have shown that higher levels of mental health literacy are associated with more positive attitudes towards professional psychological help-seeking [18–25]. For instance, a systematic review of 22 studies indicated that poor mental health literacy was a significant barrier to developing positive attitudes towards professional psychological help-seeking [26]. Another systematic review, including 53 studies, indicated that 96% of the research showed a strong positive relationship between mental health literacy and professional psychological help-seeking attitudes among young people [27]. In addition, research conducted in Korea has shown that the improvement of mental health literacy could improve the individual’s professional psychological help-seeking attitudes [23].

However, for mainland Chinese residents, the relationship between mental health literacy and professional psychological help-seeking attitudes still needs to be clarified. Indeed, cultural factors may play a significant role in shaping professional psychological help-seeking attitudes among Chinese people. Specifically, previous research indicated that Chinese people prefer to seek help from informal sources (e.g., friends, family members, and religious communities) or relieve distress by themselves [28–31]. The culture of “losing face” and “saving face” is deeply ingrained in Chinese cultural values, which can lead to the phenomenon mentioned above. As a result, Chinese residents may avoid seeking help from professional mental health services in order to protect their own and their family’s reputations [28]. Therefore, it is necessary to explore whether and how, under cultural value, mental health literacy contributed to professional psychological help-seeking attitudes among Chinese people. Understanding these complex factors can help develop effective interventions and strategies to increase the likelihood of Chinese individuals seeking professional help for mental health issues.

### Mental health literacy, psychological help-seeking stigma, and professional psychological help-seeking attitudes

Another factor that has been indicated to be related to professional psychological help-seeking attitudes is psychological help-seeking stigma. Psychological help-seeking stigma refers to the demeaning and insulting labels placed on individuals or groups seeking professional psychological help [32, 33], consisting of public stigma and self-stigma [34]. Specifically, public stigma emphasizes the attitudes of the public towards people with mental illness at the social level, and self-stigma refers to the internalization of stigma when people with mental illness feel public stigma [35].

The ABC model of attitudes posits that an individual’s affection could affect attitudes and could be affected by cognitive factors [17, 36]. Previous research has indicated that stigma has an emotional component as stigma implies public fear, anxiety, and anger, and stigmatized individuals may feel guilt, embarrassment, and self-blame [9, 37–39]. Thus, according to the ABC model, psychological help-seeking stigma may serve as a mediator between mental health literacy and professional psychological help-seeking attitudes.

Based on the ABC model of attitudes, the mediation role of psychological help-seeking stigma between mental health literacy and professional psychological help-seeking attitudes has been indicated in previous research. On the one hand, some research has shown that psychological help-seeking stigma is closely related to professional psychological help-seeking attitudes [40–44]. For example, a meta-analysis demonstrated that stigma related to professional mental health services was directly associated with negative attitudes toward professional psychological help-seeking [42]. In addition, a cross-sectional study conducted in Hong Kong indicated the negative relationship between self-stigma and attitudes toward seeking professional psychological help [41]. Recently, a systematic review showed that a significant barrier to accepting mental health services among Chinese adults is stigmatization [30]. On the other hand, the negative association between mental health literacy and psychological help-seeking stigma also had been confirmed broadly [45]. For example, a program conducted in athletics showed that the improvement of mental health literacy (e.g., knowledge and belief about mental disorders) could destigmatize the psychological help-seeking stigma [45]. Additionally, Yin et al. found a negative relationship between mental health literacy and public devaluation and discrimination of people suffering from mental health problems among 1775 Chinese people [46].

Recently, the mediation role of stigma between mental health literacy and attitudes toward seeking psychological help has been confirmed initially among Korean college students [23]. Chinese culture and Korean culture, both belonging to the Asian cultural circle, share certain cultural characteristics and traditions [23]. Thus, we hypothesize that psychological help-seeking stigma mediates the relationship between mental health literacy and professional psychological help-seeking attitudes.

### Mental health literacy, distress disclosure, and professional psychological help-seeking attitudes

Professional psychological help-seeking attitudes are also affected by distress disclosure. Distress disclosure refers to the individuals’ willingness to confide and express their negative emotions and feelings to others instead of keeping them to themselves [47].

The ABC model of attitudes also emphasizes the importance of behavioral tendency. Specifically, when individuals express the endorsement of a behavioral statement, it means that the possibility they would engage in the overt action is significantly increased, and that is to say, the attitudes of relevant behavior (e.g., seeking professional psychological help) are positive [16, 36]. Therefore, if individuals are more willing to disclose their distress, it could improve their professional psychological help-seeking attitudes. Additionally, according to the Health Disclosure Decision-Making Model (DD-MM) [48], when deciding whether to disclose personal distress and negative emotion, individuals first need to have a certain sensitivity to their mental health problems, which means mental health literacy is the prerequisite for distress disclosure [49]. Mental health literacy may increase distress disclosure. Hence, based on the above two theories, distress disclosure could mediate the relationship between mental health literacy and professional psychological help-seeking attitudes.

Empirical research has consistently shown a strong relationship between mental health literacy, distress disclosure, and professional psychological help-seeking attitudes. On the one hand, several studies have reported a positive correlation between the level of distress disclosure and professional psychological help-seeking attitudes [50–54]. For example, Schlechter et al. found that Syrian refugees perceived the overt expressing of negative emotions related to displacement and resettlement as a sign of weakness and insufficiency of strength, which impaired their ability to express pain and distress [52]. It contributed to their negative attitudes toward seeking professional psychological help. In addition, a cross-sectional study of Dutch university students found that distress disclosure was a significant predictor of professional psychological help-seeking attitudes [51]. On the other hand, there is considerable literature demonstrating that health literacy (e.g., sufficient knowledge) is linked with distress disclosure [51, 55]. For instance, some research posits that numerous patients felt their disease was too complicated and that they did not have enough knowledge to explain it. Therefore, although they had the intention of disclosure, they would still choose to be silent [55–57]. A thematic analysis conducted by Rasmussen et al. also suggested that adolescents’ own mental health literacy was one of the overarching factors of distress disclosure [49].

Overall, associations from theories and prior research indicated that distress disclosure could play a mediation role in explaining the relationship between mental health literacy and professional psychological help-seeking attitudes.

### The potential chain mediating models

How does mental health literacy work on professional psychological help-seeking attitudes when considering both emotion factors (i.e., psychological help-seeking stigma) and behavior tendency factors (i.e., distress disclosure)? Norman broadened the content of the theory on the basis of the ABC model of attitudes, proposing that individuals with affective-cognitive solid consistency are more likely to engage in attitude-relevant behavior [58]. It means that the association between mental health literacy and psychological help-seeking stigma may predict levels of distress disclosure. Moreover, DD-MM proposed that individuals will deeply consider the risks of disclosing their distress before doing so [48]. As psychological help-seeking stigma is closely related to negative comments from others, it may act as a significant risk factor and predict the possibility of an individual’s distress disclosure [49]. Therefore, it is possible that psychological help-seeking stigma and distress disclosure may play mediating chain roles between mental health literacy and professional psychological help-seeking attitudes.

However, previous studies have yielded conflicting conclusions regarding the relationship between stigma and distress disclosure. Specifically, Greenland et al. conducted a study of high school students in the United Kingdom [59]. They suggested that stigma could predict distress disclosure negatively, and the predictive effect was robust in different genders. However, a study of Dutch university students indicated that the predictive effect of stigma on distress disclosure is insignificant [51]. Moreover, Sharma and Thomas reported a positive relationship between public stigma and distress disclosure among Indian college students, suggesting that higher levels of public stigma were associated with greater disclosure of distress [53]. These findings suggest that cultural differences may contribute to the divergent findings regarding the link between psychological help-seeking stigma and distress disclosure. Thus, it is necessary to clarify the relationship between psychological help-seeking stigma and distress disclosure among Chinese people.

### The current study

Overall, higher mental health literacy, lower psychological help-seeking stigma, and higher distress disclosure are associated with more positive attitudes toward seeking professional psychological help, according to the ABC model of attitudes and DD-MM [19–21, 23, 24, 40, 41, 43, 50–53]. Previous research has shown that higher mental health literacy is associated with lower stigma [45] and higher distress disclosure [49, 51, 55]. Additionally, psychological help-seeking stigma and distress disclosure may play mediating roles in the association between mental health literacy and professional psychological help-seeking attitudes.

**Fig. 1:**
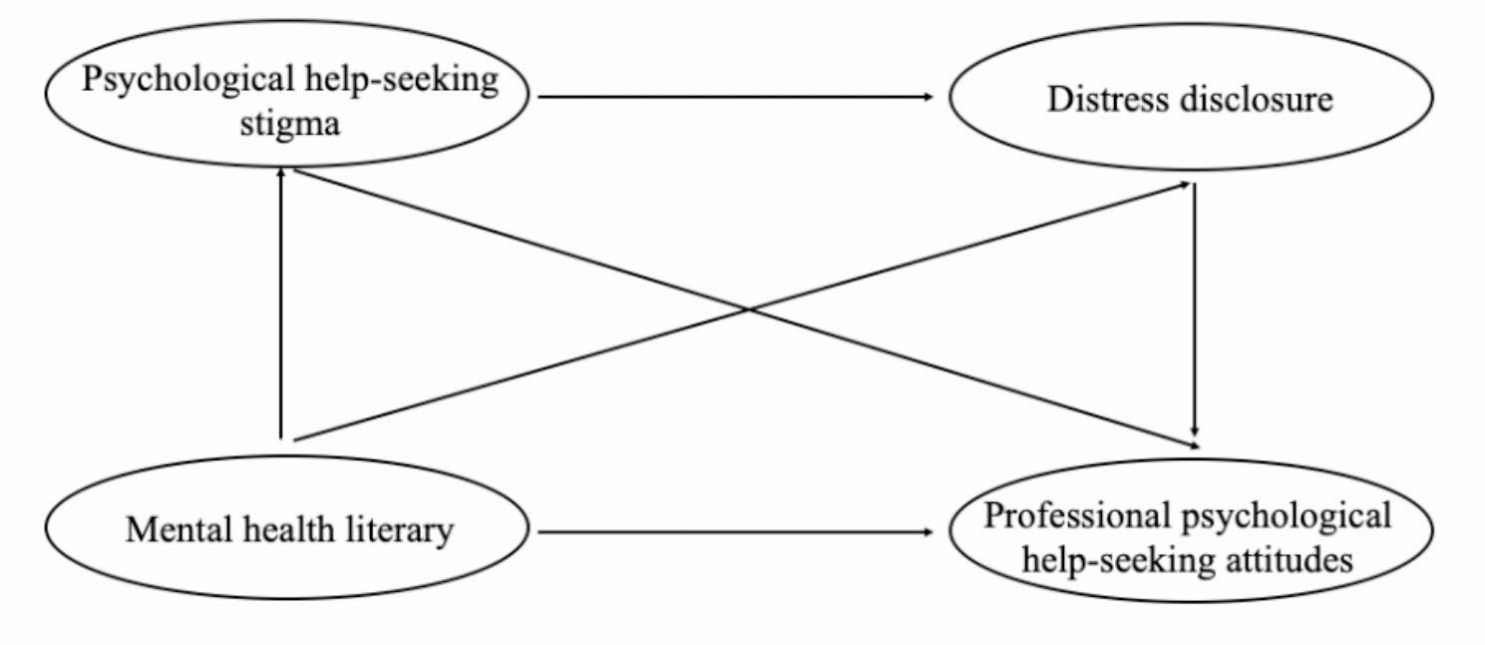
The hypothesized model of mental health literacy, psychological help-seeking stigma, distress disclosure and professional psychological help-seeking attitudes

Based on the connections discussed above, we collected data from Chinese residents to investigate a model that explains the link between mental health literacy and professional psychological help-seeking attitudes through the mediating effects of and psychological help-seeking stigma and distress disclosure. More specifically, we formulated the following hypotheses (see Fig. 1):

Hypothesis 1: Psychological help-seeking stigma would mediate the connection between mental health literacy and professional psychological help-seeking attitudes. Hypothesis 2: Distress disclosure would mediate the connection between mental health literacy and professional psychological help-seeking attitudes. Hypothesis 3: Together, psychological help-seeking stigma and distress disclosure might play mediating chain roles in the association between mental health literacy and professional psychological help-seeking attitudes.

## Methods

### Participants

We conducted a cross-sectional study of Chinese residents using convenient sampling from seven regions of China. Participants completed the questionnaire online after providing informed consent. Initially, 862 people participated in this study. However, participants who partially completed questionnaires were excluded. The final sample included 827 participants (230 males and 597 females), with a mean age of 26.019 ± 5.592 years. The response rate was 95.94%. The study was approved by the Research Ethics Committee of Beijing Forestry University.

### Measures

#### Mental health literacy (MHL)

Mental health literacy (MHL) was assessed using the recognition of disorders subscale (with eight items) of the Chinese version of The Mental Health Literacy Scale (MHLS) [60, 61]. Participants were asked to rate their agreement on a 4-point scale (ranging from 1 = very unlikely to 4 = very likely) to statements such as “To what extent do you think it is likely that Personality Disorders are a category of mental illness.” Higher scores indicate greater mental health literacy. The recognition of disorders subscale of MHLS has been validated in China and demonstrated good reliability and validity [62]. In this study, the recognition of disorders subscale of MHLS had good internal consistency (Cronbach’s α = 0.829). Additionally, the confirmatory factor analyses of the scale supported the model fit indices: χ^2^/df = 4.847, CFI = 0.975, TLI = 0.959, SRMR = 0.039, and RMSEA = 0.068.

#### Psychological help-seeking stigma

The Questionnaire of Stigma for Seeking Professional Psychological Help (SSPPH) was used to assess participants’ psychological help-seeking stigma. SSPPH was revised by Hao and Liang based on the definition of stigma [32, 33], the Stigma Scale for Receiving Psychology Help (SSRPH) [63], and the Self-Stigma of Seeking Help Scale (SSOSH) [64]. The SSPPH consisted of public stigma (with five items, e.g., “It is advisable for a person to hide from people that he/she has seen a psychologist”) and self-stigma (with five items, e.g., “Seeking psychological help would make me feel less intelligent”). Participants were asked to rate their agreement with each item on a 5-point scale (ranging from 1 = strongly disagree to 5 = strongly agree). A higher score means a more substantial stigma for seeking professional psychological of residents. SSPPH has been proven well-reliability and well-validity [33]. In this study, the SSPPH showed good internal consistency (Cronbach’s α = 0.844). Additionally, the confirmatory factor analyses of the scale supported the model fit indices: χ^2^/df = 4.328, CFI = 0.970, TLI = 0.952, SRMR = 0.043, and RMSEA = 0.063.

#### Distress disclosure

Distress Disclosure Index (DDI) was used to measure the tendency of participants to disclose or conceal their psychological distress. The DDI questionnaire consists of 12 items and was developed by Kahn and Hessling [65] and later revised by Li [66]. An example of an item from the DDI questionnaire is “When something unpleasant happens to me, I often look for someone to talk to”. Participants were asked to rate their responses on a 5-point scale (ranging from 1 = strongly disagree to 5 = strongly agree). A higher score on the DDI indicates a greater tendency to disclose psychological distress. The DDI questionnaire has been found to be reliable and valid in previous studies [67]. In the current study, the DDI questionnaire demonstrated good internal consistency (Cronbach’s α = 0.913). Additionally, the confirmatory factor analyses of the scale supported the model fit indices: χ^2^/df = 4.381, CFI = 0.964, TLI = 0.952, SRMR = 0.032, and RMSEA = 0.064.

#### Professional psychological help-seeking attitudes

A Chinese version of the Attitude Toward Seeking Professional Psychological Help-Short Form (ATSPPH-SF) with ten items was used to assess the residents’ attitudes toward professional psychological help-seeking [68]. ATSPPH-SF consisted of openness to seeking professional help (with three items, e.g., “Counseling is an ineffective approach for resolving emotional problems”), value in seeking professional help (with four items, e.g., “When I have emotional problems, I am likely to seek professional psychological help to resolve them”) and preference to cope on one’s own (with three items, e.g., “I should solve my psychological problems by myself instead of counseling”). Participants rated their responses on a 4-point scale ranging from 1 = strongly disagree to 4 = strongly agree. A higher score indicates more positive attitudes towards professional psychological help-seeking. ATSPPH-SF has been proven well-reliability and well-validity [2, 41]. In this study, ATSPPH-SF showed good internal consistency (Cronbach’s α = 0.777). Additionally, the confirmatory factor analyses of the scale supported the model fit indices: χ^2^/df = 3.584, CFI = 0.957, TLI = 0.936, SRMR = 0.035, and RMSEA = 0.056.

#### Covariates

In the current study, age, gender (1 = Male, 2 = Female), and education (1 = Middle school and below, 2 = High school, 3 = Junior college, 4 = Undergraduate, 5 = Postgraduate) were included as covariates. Because prior research indicated that age, gender, and education are statistically significantly associated with mental health literacy [9, 69], stigma [51, 69, 70], distress disclosure [50, 51, 69, 71, 72], and professional psychological help-seeking attitudes [51, 69].

### Statistical analyses

In the current study, IBM SPSS Statistics 26 version and Mplus 8.0 were used to test the hypothesized model. First, descriptive analyses and Pearson correlation analyses were conducted by SPSS 26.0. Then, Mplus 8.0 was used to evaluate the mediating chain roles of psychological help-seeking stigma and distress disclosure in the relations between mental health literacy and professional psychological help-seeking attitudes after controlling for age, gender, and education. Bootstrapping of regression estimates with 5000 samples and a 95% confidence interval was conducted. The effect is viewed as significant if zero is not included in the 95% confidence interval. In addition, cutoff values of indices of fit were used, including the χ^2^/*df* ratio, the Tucker-Lewis index (TLI), the Comparative Fit Index (CFI), Standardized Root Mean Square Residual (SRMR), and the Root Mean Square Error of Approximation (RMSEA).

## Result

### Demography

Sample demographic characteristics are shown in Table 1. Eighty hundred twenty-seven (597 females, 230 males) aged 16 to 59 years (*M* = 26.019, *SD* = 5.592) participated in this study. In this research, 262 people (31.7%) reported that they had sought professional psychological counseling due to psychological distress.

**Table 1:** Descriptive of socio-demographic. (*N* = 827)

	Total *N* (%)
**Gender**	
Male	230 (27.8)
Female	597 (72.2)
**Education**	
High school and below	46 (5.5)
Junior college	50 (6.0)
Undergraduate	508 (61.4)
Postgraduate	223 (27.0)
**Seek professional psychological counseling**	
Yes	262 (31.7)
No	565 (68.3)

### Preliminary and correlation analyses

The results of the unrotated factor analysis indicated that eight factors with characteristic roots were more than 1, and the first principal factors explained 22.361% of the variance. Therefore, there is no serious common method bias in this study.

As shown in Table 2, bivariate correlation analysis revealed that mental health literacy was positively associated with distress disclosure (*r* = 0.141, *p <* 0.001) and professional psychological help-seeking attitudes (*r* = 0.287, *p <* 0.001) and negatively associated with psychological help-seeking stigma (*r* = -0.102, *p <* 0.01). Psychological help-seeking stigma was negatively associated with distress disclosure (*r* = -0.367, *p <* 0.001) and professional psychological help-seeking attitudes (*r* = -0.469, *p <* 0.001). Distress disclosure was positively associated with professional psychological help-seeking attitudes (*r* = 0.358, *p <* 0.001).

**Table 2:** Correlations analysis between variables

	*M*	*SD*	1	2	3	4
1. Mental health literacy	2.642	0.548	1			
2. Psychological help-seeking stigma	2.111	0.647	-0.102^**^	1		
3. Distress disclosure	3.502	0.806	0.141^***^	-0.367^***^	1	
4. Professional psychological help-seeking attitudes	2.942	0.374	0.287^***^	-0.469^***^	0.358^***^	1

*Note*. *N* = 323, ^**^p < 0.01, ^***^p < 0.001

### Mediation analyses

**Fig. 2:**
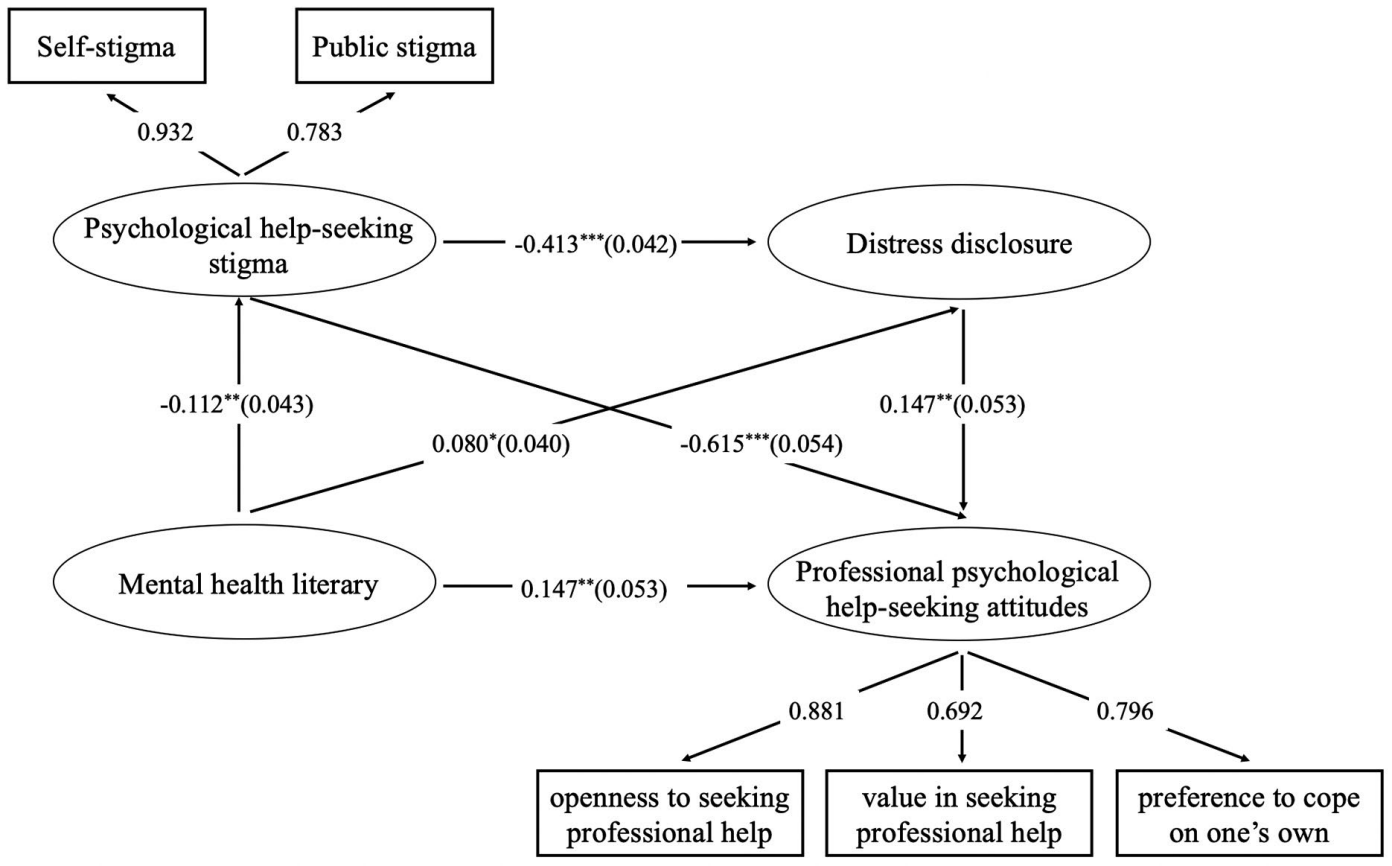
The final chain mediation model Note:^*^p < 0.05, ^**^p < 0.01, ^***^p < 0.001. Controlling for age, gender, and education

Structural equation modeling was employed to examine the mediating role of psychological help-seeking stigma and distress disclosure in the association between mental health literacy and professional psychological help-seeking attitudes. The model revealed a satisfactory fit to the data, χ^2^ (830) = 1950.605, *p* < 0.001, χ^2^/ df = 2.35, CFI = 0.916, TLI = 0.909, SRMR = 0.059, RMSEA = 0.040. After controlling for age, gender, and education, the results of the mediation effect analysis are in Fig. 2 and Table 3. Specifically, the direct pathway from mental health literacy to psychological help-seeking stigma (β = -0.112, *p* < 0.01), distress disclosure (β = 0.080, *p* < 0.05), and professional psychological help-seeking attitudes (β = 0.185, *p* < 0.001), from psychological help-seeking stigma to distress disclosure (β = -0.413, *p* < 0.001) and professional psychological help-seeking attitudes (β = -0.615, *p* < 0.001), and from distress disclosure to professional psychological help-seeking attitudes (β = 0.147, *p* < 0.01) were significant. Of the indirect effects, the path from mental health literacy to professional psychological help-seeking attitudes through psychological help-seeking stigma was significant (β = 0.012, 95% CI = 0.019 to 0.125). The path from mental health literacy to professional psychological help-seeking attitudes via distress disclosure was significant (β = 0.069, 95% CI = 0.001 to 0.031). Further, the sequential pathway from mental health literacy to professional psychological help-seeking attitudes via psychological help-seeking stigma and distress disclosure was significant (β = 0.007, 95% CI = 0.001 to 0.016). These chain mediation models explained a significant amount of variance in professional psychological help-seeking attitudes (*R*^2^ = 0.560).

**Table 3:** Chain mediation model

Pathway	β	*SE*	*Boot LLCI*	*Boot ULCI*
Direct effect				
Mental health literacy → Psychological help-seeking stigma	-0.112	0.043	-0.195	-0.029
Mental health literacy → Distress disclosure	0.080	0.040	0.001	0.158
Mental health literacy → Professional psychological help-seeking attitudes	0.185	0.043	0.103	0.271
Psychological help-seeking stigma → Distress disclosure	-0.413	0.042	-0.492	-0.330
Psychological help-seeking stigma → Professional psychological help-seeking attitudes	-0.615	0.054	-0.721	-0.511
Distress disclosure → Professional psychological help-seeking attitudes	0.147	0.053	0.038	0.245
Indirect effects				
Mental health literacy → Psychological help-seeking stigma → Professional psychological help-seeking attitudes	0.012	0.007	0.019	0.125
Mental health literacy → Distress disclosure → Professional psychological help-seeking attitudes	0.069	0.027	0.001	0.031
Mental health literacy → Psychological help-seeking stigma → Distress disclosure → Professional psychological help-seeking attitudes	0.007	0.004	0.001	0.016

*Note.* β = standardized regression coefficient; SE = standard error; LCI = lower bound of 95% confidence interval; UCI = upper bound of 95% confidence interval

## Discussion

Based on the ABC model of attitudes and DD-MM, the current study explored whether psychological help-seeking stigma and distress disclosure, two crucial factors indicated in the literature on mental health services [30, 41, 42, 51, 52], could explain the relationship between mental health literacy and professional psychological help-seeking attitudes among Chinese residents. Our study found that psychological help-seeking stigma and distress disclosure played mediating chain roles between mental health literacy and professional psychological help-seeking attitudes (i.e., mental health literacy → psychological help-seeking stigma → distress disclosure → professional psychological help-seeking attitudes). Above conclusion contributes to an expanding body of literature that shows how mental health literacy contributes to attitudes toward seeking professional psychological help, thus enriching the relevant research within the Chinese cultural context.

### The direct effect of mental health literacy on professional psychological help-seeking attitudes

Consistent with our hypothesis, there was a direct relationship between mental health literacy and professional psychological help-seeking attitudes among Chinese residents, which is consistent with the prior conclusion [18–20].

There are several explanations for this finding. First, enhanced mental health literacy enables individuals to gain a better understanding of mental health professionals and the established norms within the mental health services field. As a result, individuals become less concerned about breaching confidentiality regulations and gradually develop trust in formal resources [73]. This increased trust and familiarity contribute to a greater openness to seeking professional psychological help. Second, as individuals acquire knowledge about mental health continuously, their attributions regarding psychological problems undergo a shift. Specifically, individuals come to realize that mental health issues are not solely caused by external stressors, biological factors, or personal weaknesses [74]. This shift in perception highlights the necessity of seeking professional psychological help, as individuals recognize the value and importance of engaging in such help-seeking behaviors. Third, higher levels of mental health literacy empower individuals to adopt more effective coping strategies when confronted with mental health challenges and enable them to adaptively address these issues [75]. Consequently, individuals are more inclined to seek professional psychological support rather than relying solely on self-directed solutions. Taken together, a direct positive relationship between mental health literacy and professional psychological help-seeking attitudes was found.

### The mediation effect of psychological help-seeking stigma

The study suggested that psychological help-seeking stigma played a mediating role between mental health literacy and professional psychological help-seeking attitudes, which is consistent with the conclusion of the prior studies [23].

According to Rosenberg and Hovland [17], as mental health literacy increases, psychological help-seeking stigma tends to decrease, thereby influencing and transforming individuals’ professional psychological help-seeking attitudes. Specifically, a lack of knowledge regarding mental illness, prevention strategies, and the promotion of one’s own mental well-being can reinforce individuals’ existing stereotypes about mental illness, subsequently reinforcing cognitive stigma associated with seeking professional psychological help (i.e., public stigma). Consequently, individuals may exhibit greater resistance towards seeking professional psychological help (Xu et al., 2021). Furthermore, lower levels of mental health literacy have been found to be closely linked to a stronger identification with Confucian traditional culture [62, 76]. Confucianism attributes mental health issues to individual morality and places emphasis on concepts such as preserving face and familism [77]. As a result, individuals who seek mental health services may experience a sense of shame (i.e., self-stigma) because openly discussing mental health problems outside the family is viewed as a sign of personal weakness and can bring dishonor to the family’s reputation [78]. Consequently, individuals may actively avoid seeking help from others.

### The mediation effect of distress disclosure

Consistent with our expectations, distress disclosure mediated the association between mental health literacy and professional psychological help-seeking attitudes, which confirmed the ABC model of attitudes and the Health Disclosure Decision-Making Model [16, 36, 48]. People with insufficient mental health knowledge may attribute psychological symptoms to social-economic conditions (e.g., job stress, low income), which leads them to falsely believe that the mental health symptoms are temporary and will disappear over time without disclosing distress and seeking mental health services [9, 79]·. Moreover, a lower level of mental health literacy could limit the individual’s cognition of the necessity of distress disclosure and professional psychological services [30]. It means that even if people recognize that negative emotions or adverse symptoms are caused by mental health problems, they will insist that they can solve it on their own without expressing distress and seeking help from professionals [30, 80, 81]. Therefore, distress disclosure mediated the relationship between mental health literacy and professional psychological help-seeking attitudes.

### The chain mediating models

Further clarification regarding the relationship between mental health literacy and professional psychological help-seeking attitudes can be attributed to the chain mediation effect of psychological help-seeking stigma and distress disclosure, aligning with the initial hypothesis. This explanation draws upon the ABC model of attitudes [58] and DD-MM [48]. As previously mentioned, an increase in mental health knowledge diminishes individuals’ stigma surrounding seeking mental health services [76], which enabled them to view the impact of distress disclosure to others in a neutral or positive way. Thus, the perceived pressure associated with distress disclosure diminishes. These cognitive, emotional, and behavioral factors collectively contribute to the change in attitudes.

However, the conclusion was contrary to previous research conducted by Sharma and Thomas [53]. In Sharma and Thomas, the relationship between stigma and distress disclosure was significantly positive among Indian university students, which was unexpected [53]. The contrasting findings between the two studies may be attributed to cultural differences. Specifically, collectivist and Confucian values in China emphasize the importance of maintaining harmonious social relationships, where inappropriate behaviour can be stigmatized by the public, potentially leading to discrimination [38, 82]. Influenced by these cultural values, Chinese individuals tend to conform to social norms (e.g., Individuals should maintain social harmony rather than expressing personal opinions, emotions, and values) to avoid social exclusion [13, 46, 83]. Thus, public stigma in the Chinese context often translates into self-stigma and negatively predicts individual self-disclosure. However, while Indian residents experiencing mental health issues tend to be more sensitive to the prejudice and discrimination associated with public stigmatization, this heightened sensitivity not only acts as a deterrent to internalizing public stigma but also facilitates their ability to articulate and express their emotional distress [84]. Consequently, the negative relationship between stigma and distress disclosure was not found in the Indian context.

### Limitations and outlook

This study has several limitations that should be acknowledged. First, the study design was cross-sectional, which hinders establishing causal relationships between mental health literacy, psychological help-seeking stigma, distress disclosure, and professional psychological help-seeking attitudes. Therefore, future research can explore the causal relationship of each variable using longitudinal design or experimental design (e.g., exploring whether the interventions towards increasing mental health literacy could reduce the stigma of psychological help-seeking and improve the level of distress disclosure) further. Second, the sample in this study was obtained through snowball sampling on social platforms, resulting in an unbalanced gender distribution, with over 70% of participants being women. This issue of unbalanced gender representation has been observed in previous studies as well, limiting the external validity of the findings [41]. Subsequent studies should employ more systematic and scientific sampling methods, increase the sample size, and strive to achieve a balanced gender ratio to enhance the external validity of relevant research. Third, the ultimate outcome of the current study is professional psychological help-seeking attitudes rather than actual help-seeking behavior. Although the relationship between attitudes and behavior has been suggested [10, 13], future studies should still investigate whether this chain mediation model continues to hold in actual professional psychological help-seeking behavior [10].

### Conclusion and implications

The current study, based on the ABC model of attitudes and the Health Disclosure Decision-Making Model, makes a valuable contribution to the existing literature on professional psychological help-seeking attitudes among Chinese individuals. It reaffirms the significance of mental health literacy, psychological help-seeking stigma, and distress disclosure in shaping these attitudes. Furthermore, the study highlights the mediating roles of psychological help-seeking stigma and distress disclosure in the relationship between mental health literacy and professional psychological help-seeking attitudes. The findings of the study have important implications for public education campaigns aimed at promoting the utilization of mental health services. For instance, professional psychologists can conduct psychoeducational sessions that focus on topics such as the causes of mental health issues, strategies for maintaining good mental health, and ways to effectively utilize professional mental health services [13]. Additionally, replacing stereotyped descriptions of people with mental disorders with factual statements on social media could also reduce the stigma of psychological help-seeking and mental illness [37].

## Data Availability

The datasets used and analyzed during the current study are available from the corresponding author on reasonable request.
